# Identification of novel CD8+ T cell epitopes in human herpesvirus 6B U11 and U90

**DOI:** 10.1002/iid3.55

**Published:** 2015-04-28

**Authors:** Mustafa Halawi, Naeem Khan, Neil Blake

**Affiliations:** 1 Institute of Infection and Global Health, University of LiverpoolLiverpool, UK; 2 Department of Clinical ImmunologySchool of Immunity and Infection, University of BirminghamBirmingham, UK

**Keywords:** HHV6B, T cells, CD8+, Epitopes, virus-infected cells

## Abstract

Human herpesvirus 6B (HHV6B) infects over 90% of the population, and normally establishes a latent infection, where episodes of reactivation are asymptomatic. However, in immunocompromised patients HHV6B reactivation is associated with high morbidity and mortality. Cellular immunotherapy has been utilised against other herpesvirus in immunocompromised settings. However, limited information on the immune response against HHV6B has hampered the development of immunotherapy for HHV6B-driven disease. In this study, we have analysed the cellular immune response against four HHV6B antigens in a panel of 30 healthy donors. We show that the base-line level of T cell reactivity in peripheral blood is very low to undetectable. A short-term reactivation step enabled expansion of T cell responses, and all donors responded to at least 1 antigen, but more commonly 3 or 4. A hierarchy of immunogenicity was determined with antigens U90 and U54 being co-dominant, followed by U11 and U39. Putative CD8+ T cell epitopes were mapped to U90 and U11, predicted to be presented in the context of HLA-A1, A29, B39 and C6. T cells reactive against these novel epitopes were able to recognise virus-infected cells. Our data is supportive of the application and on-going development of T cell immunotherapy against HHVB-driven disease in the immunocompromised host.

## Introduction

Human herpesviruses are ubiquitous pathogens that generally cause self-limited disease in healthy individuals. However, in the context of an immunosuppressed host herpesvirus infection can lead to serious, often fatal disease 1. Adoptive T cell therapy has been utilised as a therapeutic modality in immunocompromised individuals, such as recipients of hematopoietic stem cell (HSCT) or solid organ transplant (SOT), where the herpesviruses Epstein–Bar virus (EBV) and Human cytomegalovirus (HCMV) are important drivers of disease 2–9. However, this has only been possible due to the in-depth knowledge and understanding of the cellular immune response against these viruses 10,11. This is in direct contrast to another herpesvirus, the β-herpesvirus human herpesvirus 6 (HHV6), which is also a significant pathogen associated with poor outcome in situations such as HSCT and SOT 12,13.

HHV6 exists as two species, HHV6A and HHV6B, with a high level of nucleotide similarity 13–15. The epidemiology and clinical features of HHV6A infection are poorly characterised 12. By contrast, HHV6B is present worldwide with greater than 90% of the population carrying the virus 12. Primary infection with HHV6B usually occurs during infancy, and is associated with a self-limiting disease, presenting as a febrile illness or exanthema subitum 16,17. After primary infection, HHV6B establishes a life-long latent infection, and in immunocompetent individuals reactivation is generally asymptomatic 12. However, in an immunosuppressed patient, such as HSCT and SOT, reactivation of HHV6B is associated with significant clinical pathology, which includes graft versus host disease (GvHD) and encephalitis, and can lead to increased mortality 18–22. Although assumed to be important for control of HHV6B reactivation, details of the cell-mediated immune response against HHV6B are only just beginning to be elucidated. Recent studies have only just started to map and characterise the T cell responses against antigens from HHV6B. T cells have been shown to be reactive against antigens such as U11, U14, U54, U71 and U90, and HLA Class I restricted epitopes identified and mapped to HLA-A2, A3, B7 and B40 23–26. However, there is still a need to continue to fully characterise the immune responses to HHV6B and to expand the database of knowledge on T cell targets and HLA restricting elements.

In the current study, we have analysed a cohort of healthy donors for T cell responses to HHV6B antigens U90, U11, U54 and U39, representing an immediate early antigen, two viral tegument proteins and a viral envelope glycoprotein, respectively 15. In agreement with other reports, we show that base-line levels of HHV6-specific T cells in peripheral blood is very low or undetectable, but that reactive T cells can be successfully expended *in vitro* by short-term stimulation with appropriate antigenic peptides. Indeed, of 30 donors analysed all were able to mount responses to at least one of the four target antigens, with the majority of donors responding to three or all four. We identify three novel putative CD8+ T cell epitopes in U90, predicted to be restricted through HLA-A1, -A29 and -B39, and one epitope in U11, restricted through HLA-C6. Importantly, T cells reactivated with these peptides were able to recognise HHV6B-infected target cells highlighting their potential clinical utility. The continual identification and characterisation of the targets of HHV6-specific T cells is important for the future development of T cell therapies against HHV6B driven disease, and the data presented here is an important addition.

## Results

### *Ex vivo* analysis of T cell responses to HHV6B U11, U39, U54 and U90

**Figure 1:**
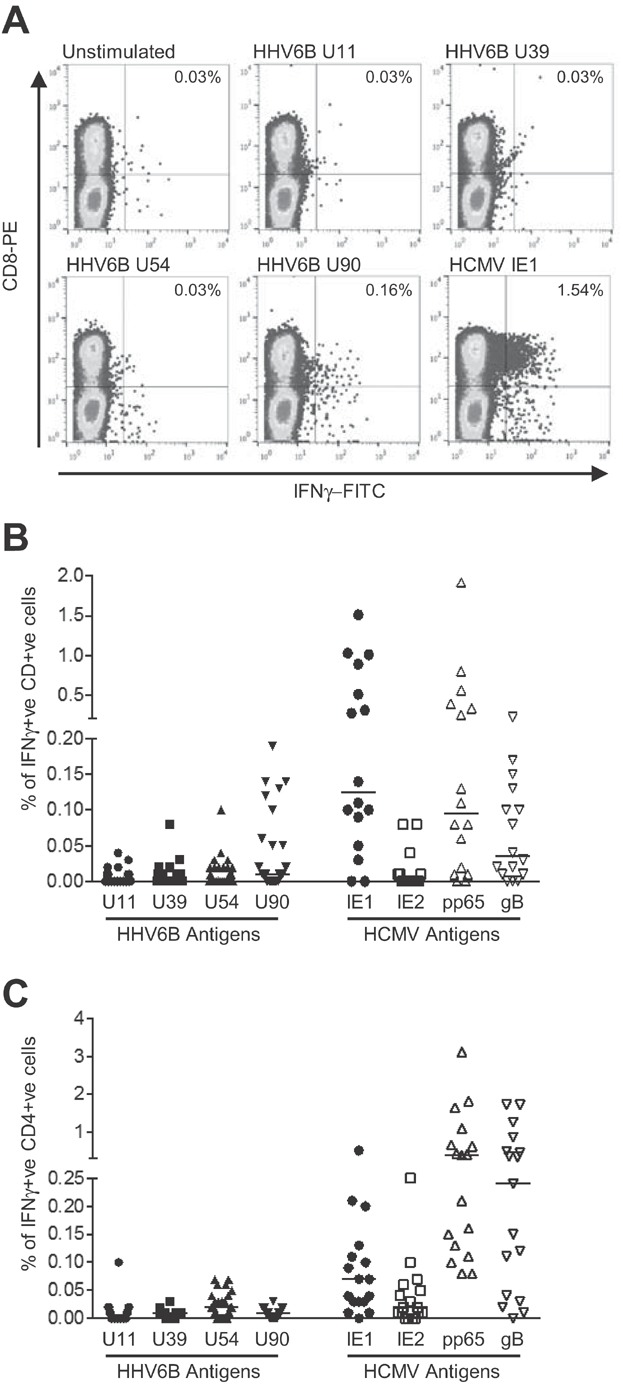
*Ex vivo* analysis of T cell responses to HHV6B antigens U11, U39, U54 and U90. T cell responses to HHV6B antigens U11, U39, U54 and U90 in peripheral blood were analysed in a panel of 30 healthy donors by ICS for IFN-γ after overnight stimulation with 15-mer PepMixes^TM^. Cells were stained with mAbs for CD8 or CD4 and IFN-γ, followed by analysis by flow cytometry. (A) A representative flow cytometry analysis for CD8+IFNγ+ responses is shown for donor HD05. The percentages of CD8+ve IFN-γ+ve T cells are shown in the top right hand quadrant. Analysis of PBMC stimulated with a PepMix^TM^ for HCMV IE1 is also shown. ConA stimulated PBMC was used as a positive control (not shown). (B) The percentage of CD8+ve IFN-γ+ve T cells for HHV6B antigen U11, U39, U54 and U90 are shown for all donors (*n* = 30). HCMV-seropositive donors (*n* = 15) were also analysed for responses to four immunogenic HCMV antigens (IE1, IE2, pp65 and gB). The median frequency is indicated with a bar. Data are shown after subtraction of the background value for unstimulated cells. (C) The same panel of donors were analysed for CD4+ IFNγ+ T cells. The percentage of positive cells are shown as in part (B).

Very little is known about which HHV6B antigens are targeted by T cells during HHV6 infection, and how immunogenic such antigens would be. Given the high degree of homology between HHV6B and a second human β-herpesvirus, HCMV, we set out to determine if T cell responses could be detected directly *ex vivo* against HHV6B antigens corresponding to known immunogenic HCMV proteins. We focused on four antigens from HHV6B, namely U11, 39, U54 and U90, corresponding to HCMV antigens pp150, gB, pp65 and IE1. PBMCs were isolated from a panel of 30 donors, with a broad range of HLA backgrounds, stimulated for 16 h with single tube 15-mer PepMixes^TM^ for each HHV6B antigen, and analysed for the frequency of CD8+ve, IFN-γ+ve and CD4+ve, IFN-γ+ve cells by ICS. A representative example of the flow cytometry analysis of HHV6 antigen-specific CD8+ve, IFN-γ+ve cells is shown for donor HD05 in Figure 1A. For this donor responses against the HHV6B antigens U11, U39 and U54 were equivalent to background unstimulated cells. A detectable response was seen against U90 (0.16%), although this was significantly lower than the representative HCMV antigen, IE1 (1.54%). Overall, for all donors the frequency of CD8+ T cells detected against the four HHV6B antigens was very low, in most cases barely above detected levels (Fig. 1B). The median values for U11, U39 and U54 were 0.00% IFNγ+ CD8+ T cells (ranges 0–0.04, 0–0.08, 0–0.1% respectively), whereas the median value for U90 was 0.01% (range 0–0.19%, *n* = 30). Only seven donors had levels detectable at 0.1% or above after subtraction of the unstimulated background, six for U90 (donors HD03, 0.1%; HD05, 0.13%; HD09, 0.19%; HD13, 0.14%; HD20, 0.14%; and HD30, 0.12%) and one for U54 (HD03, 0.1%). To ensure that this cohort of donors could respond to herpesviruses, 15 HCMV seropositive donors were also analysed for responses to four immunogenic antigens from HCMV (namely IE1, IE2, gB and pp65). Good responses were detected against IE1, pp65 and gB, with IE2 being recognised at low levels by a small number of donors in this group. Overall median values for the HCMV antigens were IE1 0.13%, IE2 0.00%, pp65 0.10% and gB 0.04% (ranges 0–1.5, 0–0.08, 0–1.9, 0–0.22% respectively, *n* = 15, Fig. 1B).

Similarly, very low levels of HHV6 antigen-specific CD4+ve, IFN-γ+ve cells were detected (Fig. 1C). The median values were U11 0% (range 0–0.01%), U39 0.01%, (0–0.03%), U54 0.02% (0–0.07%) and U90 0.01% (0–0.03%, *n* = 30). Responses to HCMV IE1, IE2, gB and pp65 were also measured, and found to be present at detectable levels in the majority of HCMV-seropositive donors tested, median values for IE1 0.07% (0–0.52%), IE2 0.02% (0–0.25%), pp65 0.4% (0.08–3.13%) and gB 0.24% (0–1.7%, *n* = 10, Fig. 1C).

### Short-term in vitro stimulation expands T cell responses against U11, U39, U54 and U90

**Figure 2:**
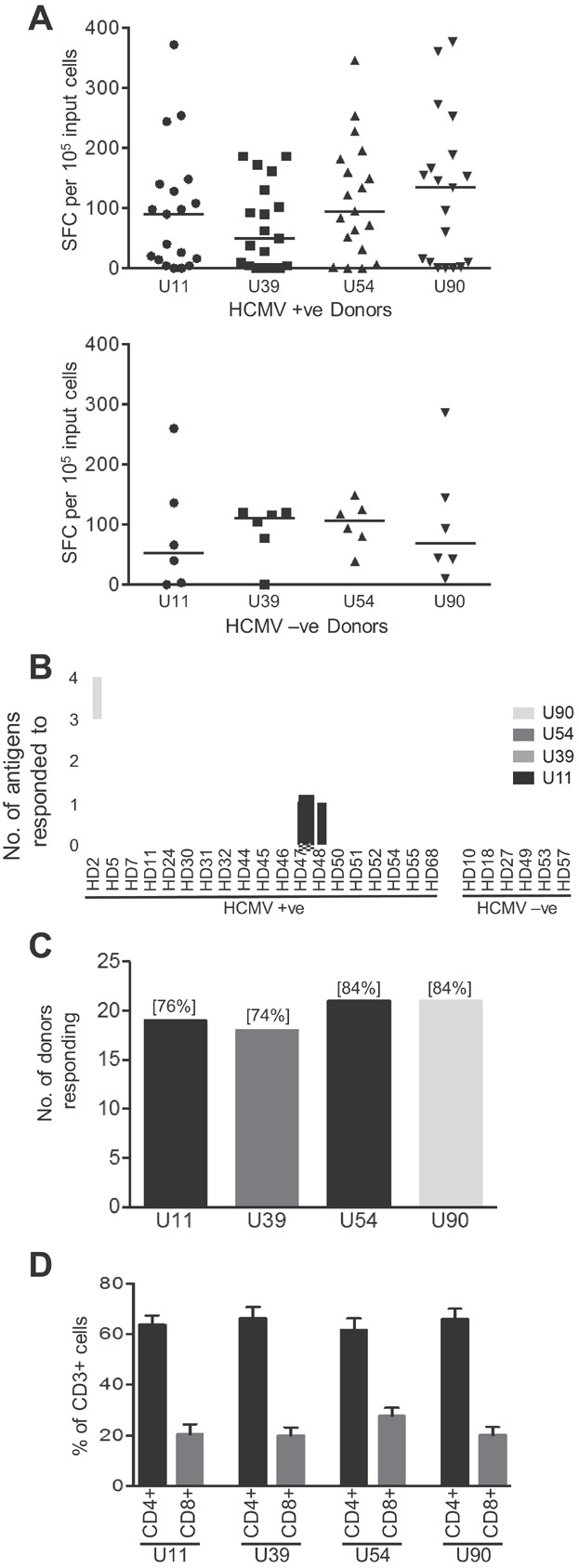
Breadth of T cell responses specific for HHV6B antigens U11, U39, U54 and U90 analysed by IFN-γ ELISPOT after a short-term *in vitro* expansion. (A) PBMC from 25 donors were stimulated with HHV6B PepMixes^TM^ and cultured for 10 days, with the addition of IL2 from day 3. The frequency of HHV6B antigen-specific T cells was evaluated by IFN-γ ELISPOT. The data are presented for 19 HCMV seropositive donors (top panel) and 6 HCMV seronegative donors (lower panel). Results are presented as IFN-γ spot forming cells (SFC)/10^5^ input cells, after subtraction of the background value for the negative control (DMSO pulsed cells), with the median value indicated with a bar. (B) The number of donors responding to each antigen is represented graphically, with the percentage responding in brackets, and (C) The number of antigens, and specific antigen, each donor responded to is shown. (D) To determine the phenotype of T cells present in PepMix^TM^ expanded populations, cells were stained for CD4+ and CD8+ cells (see Supporting Information Fig. S1). The mean frequency of CD3+ CD4+ and CD3+ CD8+ cells for each antigen PepMix^TM^ are shown (±SEM*, n* = 8).

On the assumption that T cells against these HHV6B antigens were present but at barely detectable levels *ex vivo*, we predicted that a short-term *in vitro* reaction would expand these T cells such that we could begin to characterise the responses in further detail. Using the individual 15-mer PepMixes^TM^ for U11, U39, U54 and U90, we stimulated PBMC from 25 donors for 10 days before analysis of expanded populations by direct ELISPOT using the PepMixes^TM^. Figure 2A shows the data from 19 HCMV seropositive donors (top panel) and 6 HCMV seronegative donors (lower panel). We were able to detect IFNγ+ cells by ELISPOT against all antigens. In the HCMV seropositive donors, U90 showed the highest frequency of IFNγ+ cells, with a median value of 134 SFC/10^5^ input cells (range 0–376), with median responses to U54, U11 and U39 of 95 (range 0–346), 90 (range 0–372) and 50 (range 0–186) SFC/10^5^ input cells, respectively (*n* = 19). We were able to expand HHV6B-specific T cells from the HCMV-seronegative donors supporting the conclusion that we are expanding authentic HHV6B response rather than cross-reactive HCMV T cells. There was a slight difference in the strength of responses detected in these donors, with U39 showing the strongest response with median value of 110 SFC/10^5^ input cells (range 0–120), followed by U54 106 (range 39–149), U90 69 (range 10–286) and U11 53 (range 0–260) SFC/10^5^ input cells (*n* = 6). However, when data from all donors were combined the median responses were U90 96 (range 0–376) SFC/10^5^ input cells followed by U54 95 (range 0–346), U39 77 (range 0–186) and U11 66 (range 0–372) SFC/10^5^ input cells. When analysed individually all donors responded to at least one antigen: 14 donors responded to all 4 antigens, 3 donors responded to 3 antigens, 6 responded to 2 and only 2 donors recognised only 1 (Fig. 2B). In total 21/25 donors (84%) responded to both U54 and U90, 19/25 (76%) responded to U11 and 18/25 (74%) responded to U39. Based on the approaches used here, although the overall responses were similar, a hierarchy could be established with U90 and U54 being the most immunodominant, with very little difference between them, followed by U11 and U39.

Since the IFNγ ELISPOT does not discriminate between reactive CD8+ and CD4+ T cells we next performed phenotypic analyses of our polyclonal T cell populations (see Supporting information Fig. S1). This revealed that for each PepMix^TM^ expanded T cell population, the majority of CD3+ T cells were also CD4+, with a mean frequency for U11 of 63.5 ± 10.8%; U39 66.3 ± 12.4%; U54 61.6 ± 12.9%, U90 65.9 ± 11.6% (*n* = 8). Correspondingly, lower values of CD3+CD8+ cells were seen in the expanded populations for each antigen, U11 21.5 ± 11.0%; U39 20 ± 8.9%; U54 27.9 ± 8.9%, U90 20.1 ± 9.1% (Fig. 2D).

### Identification and HLA mapping of CD8+ T cell epitopes in U90

**Figure 3:**
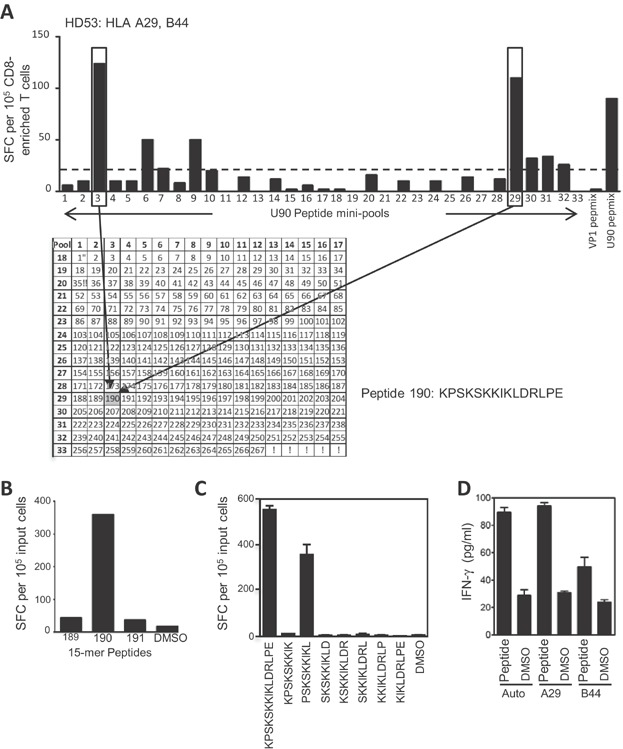
Identification of the CD8+ T cell epitope PSKSKKIKL in HHV6B U90, restricted through HLA-A29. (A) Polyclonal T cells from donor HD53 (HLA-A29, B44, C5, C16) expanded in vitro by stimulation with the U90 PepMix^TM^ were depleted of CD4+ cells before being screened by IFN-γ ELISPOT using U90 mini pools. The 33 mini pools contain 267 overlapping 15-mers representing the entire U90 protein, with each 15-mer present in two pools (shown in the grid). PepMixes^TM^ representing U90 and BK polyomavirus VP1 were used as positive and negative controls, respectively. A pool was considered positive if the response was 2.5-fold greater than background, indicated by the dashed line. Two strongly positive pools (3 and 29) are boxed and the common 15-mer, peptide 190 (KPSKSKKIKLDRLPE), highlighted by arrows. (B) HD53 U90 PepMix^TM^ reactivated T cells, depleted of CD4+ cells, were then screened in ELISPOT against the 15-mer peptide 190 on it is own, along with the flanking 15-mers 189 and 191. (C) Individual overlapping 9-mer peptides spanning peptide 190 were tested against the same expanded T cell population identifying the minimal peptide as PSKSKKIKL. Results for ELISPOT experiments are presented as IFN-γ spot-forming cells (SCF)/10^5^ input cells (±SD). (D) Autologous or partially HLA-matched target cells were pulsed with either the PSKSKKIKL 9-mer peptide or DMSO, and used as targets in T cells assays with U90 PepMix^TM^ expanded total T cells, at an E/T ratio of 10:1. T cell recognition of targets was monitored by detection of IFN-γ in culture supernatants by ELISA. Data are presented as pg/mL, and is shown as the mean of triplicate wells (±SD).

Having shown that the circulating precursor frequency of HHV6-directed T cells was low in peripheral blood, but that reactive cells could be readily expanded by a short-term stimulation *in vitro*, we next sought to identify and map individual CD8+ epitopes. Polyclonal T cell populations were expanded using the U90 PepMix^TM^ from selected donors shown to contain U90-specific responses (Fig. 2). To ensure that we would maximise the possibility of identifying CD8+ responses, expanded T cells were enriched for CD8+ T cells by depletion of CD4+ cells. The first donor analysed was donor HD53 (HLA-A29, B44, C5, C16), with a fourfold enrichment of CD8+ T cells (increase from 16% to 67%) achieved by depleting CD4+ T cells (Supporting Information Fig. S2). These CD8-enriched cells were then analysed by ELISPOT for IFNγ+ve cells by direct stimulation with 33 U90 mini pools of overlapping 15-mer peptides. The results of the mini-pool screen are shown in Figure 3A, with two strong responses identified against pool 3 and 29. Using the 2D grid representing the U90 mini pools, the peptide that is present in both of these pools was identified as peptide 190, KPSKKKIKLDRLPE (aa 766–780). The same expanded, CD8-enriched T cells were then screened against peptide 190 on its own, along with the flanking 15-mer peptides (189 and 191, Fig. 3B). This confirmed that peptide 190 was recognised by T cells from donor HD53, but neither 189 nor 191 (in agreement with the mini-pool screen data). To map the minimal peptide epitope, overlapping 9-mer peptides were synthesised spanning the peptide 190 sequence and tested by ELISPOT against CD8-enriched T cells which had been reactivated with the 15-mer peptide 190. Figure 3C shows that the minimal epitope maps to the sequence PSKSKKIKL (aa 765–773), termed PSK. To identify the HLA restricting element, autologous and partially HLA-matched target cells were pulsed with either the PSK peptide or DMSO control and used as targets in T cell assays with polyclonal T cell populations specific for PSK. Using detection of IFNγ secreted into the supernatant, we found that autologous and HLA-A29 matched targets were recognised by PSK-specific T cells, and while there was a slight recognition of B44-matched targets above background, we predict that the PSK peptide is presented to T cells in the context of HLA A29. One additional donor HLA-A29 positive donor was available and to test if this donor (HD33; HLA A11, A29, B39, B44, C9, C16) also responded to the PSK peptide, PBMCs were reactivated with the PSK peptide, cultured for 10 days in vitro before being screened by ELISPOT. A PSK-specific response was identified in this donor (Supporting Information Fig. S3), supporting this as a novel U90-derived CD8+ T cell epitope, putatively presented through HLA-A29.

**Figure 4:**
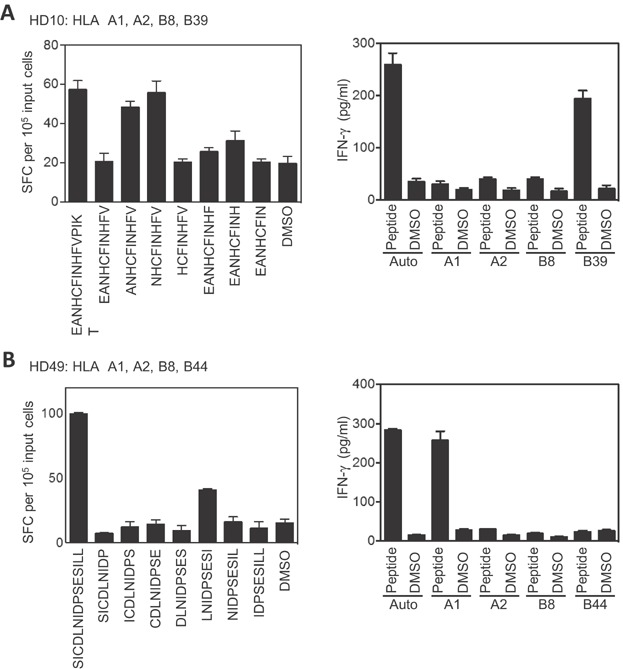
Identification of HHV6B U90 CD8+ T cell epitopes NHCFINHFV and LNIDPSESI, presented through HLA B39 and A1. (A) U90 PepMix^TM^ expanded T cells from donor HD10 (HLA-A1, A2, B8, B39) were depleted of CD4+ cells, before being tested in ELISPOT against individual overlapping 9-mer peptides spanning the 15-mer peptide 226 (EANHCFINHFVPIKT), identifying the minimal peptide as NHCFINHFV (left hand panel). Autologous or HLA-matched target cells were pulsed with either the NHCFINHFV 9-mer or DMSO, and used as targets in T cells assays with U90 PepMix^TM^ expanded T cells, at an E/T ratio of 10:1 (right hand panel). T cell recognition of targets was monitored by detection of IFN-γ in culture supernatants by ELISA. (B) U90 PepMix^TM^ expanded T cells from donor HD49 (HLA-A1, A2, B8, B44, C5, C7) were depleted of CD4+ cells, before being tested against individual overlapping 9-mer peptides spanning the 15-mer peptide 104 (SICDLNIDPSESILL), identifying the minimal peptide as LNIDPSESI (left hand panel). Autologous or partially HLA-matched target cells were pulsed with either the LNIDPSESI 9-mer or DMSO, and used as targets in T cells assays with U90 PepMix^TM^ expanded T cells, at an E/T ratio of 20:1 (right hand panel). T cell recognition of targets was monitored by detection of IFN-γ in culture supernatants by ELISA. Results for ELISPOT experiments are presented as IFN-γ spot-forming cells (SCF) per 10^5^ input cells and are shown as the mean of triplicate wells (±SD). Data from ELISA experiments are presented as pg/mL, and are shown as the mean of triplicate wells (±SD).

Analysis of two further donors (HD10 and HD49) also identified novel CD8+ T cell epitopes. A mini-pool screen of expanded T cells, enriched for CD8+ cells, from donor HD10 (HLA A1, A2, B8, B39) identified a number of potential stimulating pools. The positive pools 5 and 31 were selected for further analysis, where the common 15-mer was peptide 226, EANHCFINHFVPIKT (aa 901–915, Supporting Information Fig. S4). Using peptide 226 and overlapping peptides spanning this sequence, the response was mapped to NHCFINHFV (NHC; aa 903–911), and shown to be restricted through HLA B39 (Fig. 4A). Similar analysis of CD8-enriched cells from donor HD49 (HLA A1, A2, B8, B44, C5, C7) also identified a number of potential positive pools, with the responses to pools 2 and 24 selected for further analysis. The intersect between these pools was the 15-mer peptide 104, SICDLNIDPSESILL (aa 413–427, Supporting Information Fig. S4). Using peptide 104 and overlapping 9-mer peptides, the response was mapped to LNIDPSESI (LNI; aa 417–425), and shown to be restricted through HLA A1 (Fig. 4B).

Our analysis also confirmed responses to previously published epitopes as shown in Supporting Information Figure S5, where CD8-enriched T cells from donor HD57 (HLA A2, B40(60), B50, C6, C10) were shown to recognize the recently published B40-restricted peptides VEESIKEIL (VEE; aa 39–47) and FESLLFPEL (FES; aa 57–67) 25. (Supporting Information Fig. S5A and B). Two additional B40-positive donors (HD05 and HD30) were also shown to have responses targeting these peptides (Supporting Information Fig. S5C).

### Identification and HLA mapping of a CD8+ T cell epitope in U11

**Figure 5:**
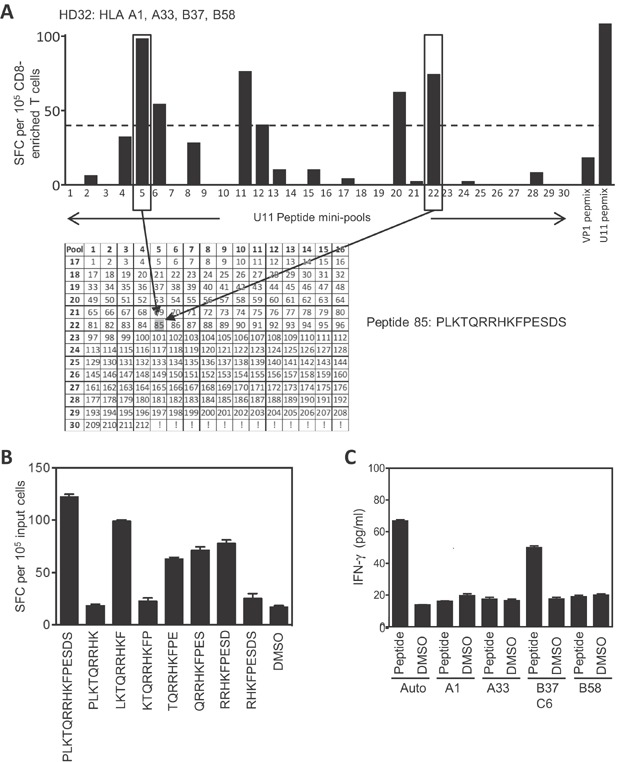
Identification of the CD8+ T cell epitope LKTQRRHKF in HHV6B U11, restricted through HLA-C6. (A) Polyclonal T cells from donor HD32 (HLA-A1, A33, B37, B58, C6, C9) expanded in vitro by stimulation with the U11 PepMix^TM^ were depleted of CD4+ cells before being screened by IFN-γ ELISPOT using U11 mini-pools. The 30 mini pools contain 212 overlapping 15-mers representing the entire U11 protein, with each 15-mer present in two pools (shown in the grid). PepMixes^TM^ representing U11 and BK polyomavirus VP1 were used as positive and negative controls respectively. A pool was considered positive if the response was 2.5-fold greater than background, indicated by the dashed line. Two strongly positive pools (5 and 22) are boxed and the common 15-mer, peptide 85 (PLKTQRRHKFPESDS), highlighted by arrows. (B) Individual overlapping 9-mer peptides spanning the 15-mer peptide 85 were tested against the same expanded T cell population identifying the minimal peptide as LKTQRRHKF. Results for ELISPOT experiments are presented as IFN-γ spot-forming cells (SCF)/10^5^ input cells, are shown as the mean of triplicate wells (+/−SD). (C) Autologous or partially HLA-matched target cells were pulsed with either the LKTQRRHKF 9-mer peptide or DMSO, and used as targets in T cells assays with U11 PepMix^TM^ expanded total T cells, at an E/T ratio of 10:1. T cell recognition of targets was monitored by detection of IFN-γ in culture supernatants by ELISA. Data are presented as pg/mL, and are shown as the mean of triplicate wells (±SD).

We applied the same strategy to mapping CD8+ T cell responses in HHV6B U11. Polyclonal T cell populations were expanded from donor HD32 (HLA-A1, A33, B37, B58, C6, C9), using the U11 PepMix^TM^, enriched for CD8+ cells and analysed by ELISPOT for IFNγ+ve cells by stimulation with 30 U11 mini-pools of overlapping 15-mer peptides (Fig. 5A). A number of responses were identified, with two strong responses identified against pool 5 and 22 selected for further analyses. The intersect of these pools maps to 15-mer 85, PLKTQRRHKFPESDS (aa 337–351, Fig. 5A). This 15-mer was recognised by T cells, as was the 9-mer LKTQRRHKF (aa 338–346), termed LKT (Fig. 5B). Responses were also seen against other 9-mers (e.g. QRR and RRH), suggesting that this 15mer may contain a second independent epitope (Fig. 5B), but this was not investigated further. LKT-specific T cells were then shown to recognise peptide-pulsed target cells carrying both HLA-B37 and -C6 alleles (Fig. 5C). Based on the peptide binding motif of these alleles, where there is strong preference for a basic anchor residue at position 2 for HLA-C6 as opposed to an acidic residue for B37, we propose the HLA restricting element of LKT is HLA-C6.

### HHV6B U90 and U11-specific T cells recognise virus-infected cells

**Figure 6:**
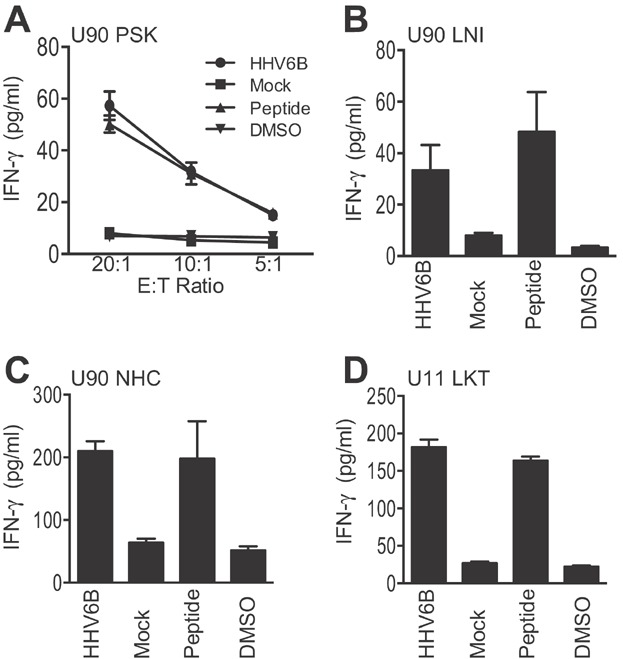
HHV6B U90- and U11-specific T cells recognise virus-infected target cells. (A) Polyclonal PSK-specific T cell effectors generated by reactivation of donor HD53 PBMC with the U90 9-mer peptide PSK were tested in T cell assays against autologous PHA blasts infected with HHV6B strain Z29 or mock infected, at the indicated E:T ratios. Control targets PHA blasts pulsed with either the PSK peptide or DMSO. (B, C and D) Polyclonal T cell effectors generated by reactivation of donor PBMC with the individual 9-mer peptides U90 9-mer NHC and LNI, and U11 LKT were tested in T cell assays against autologous PHA blasts infected with HHV6B strain Z29 or mock infected. Control targets PHA blasts pulsed with the individual 9-mer peptide or DMSO. T cells were used at an E/T ratio of 10:1. T cell recognition of target cells was determined by measuring release of IFN-γ in to the supernatant by ELISA. Results are the mean of experimental triplicates (±SD) and expressed as pg/mL.

With the ultimate objective of using these cells clinically, it was important to confirm that these new HHV6B-specific T cell epitopes could recognise endogenously expressed antigen. Thus, T cell populations were expanded using individual 9-mers representing U90 PSK, LNI and NHC, and U11 LKT and the expanded T cells were tested for recognition of HHV6B-infected autologous cells lines. Figure 6A shows the data for U90 PSK, where there is clear recognition of HHV6B-infected cells to levels equivalent to peptide pulsed targets. Staining of target cells confirms that they are efficiently infected with HHV6B virus (Supporting Information Fig. S6). Target cell recognition for LNI, NHC and LKT effectors is shown in Figure 6B–D, with all T cell lines recognising HHV6B virus-infected cells to similar levels as the peptide-pulsed controls. Whilst the expanded T cell cultures used here will undoubtedly contain other non-HHV6B specific populations of T cells, we do not believe that they contribute to the recognition observed.

## Discussion

HHV6B reactivation is a significant clinical problem after HSCT and SOT, and is associated with GvHD, encephalitis and increased mortality. Manipulation of cellular immune responses against other herpesviruses, such as EBV and HCMV, has proved to be successful in treating clinical disease driven by reactivation of these viruses in HSCT and SOT patients. This suggests that T cell immunotherapy should also be considered as a treatment option for patients with HHV6B reactivation after transplant. Currently, this is limited by a paucity of detailed information on the cellular immune response to this virus, such that although HHV6B is known to induce immune responses, there is little detailed information on target antigens, T cell epitopes or HLA restriction elements. This detail is a key requirement to understand the role of cellular immunity in controlling HHV6B reactivation, and will feed into development of future immunotherapy against this virus. In this report, we have investigated the T cell response to HHV6B infection in a group of healthy individuals, and mapped four putative novel CD8+ antigenic peptides to U90 and U11 predicted to be presented in the context of HLA alleles A1, A29, B39 and C6 (Table1).

**Table:** Summary of putative CD8+ T cell epitopes identified in HHV6B U90 and U11

HHV6B antigen	15-mer peptides recognised	Sequence^a^	Amino-acids^a^	Putative HLA restriction	Recognition of virus-infected cells
U90	SICDLNIDPSESILL	LNIDPSESI	417-425	A1	Yes
	KPSKKKIKLDRLPE	PSKSKKIKL	758-766	A29	Yes
	EANHCFINHFVPIKT	NHCFINHFV	903-911	B39	Yes
					
U11	PLKTQRRHKFPESDS	LKTQRRHKF	337-345	C6	Yes

a Amino-acid sequence and positions are based on HHV6B strain Z29 15.

We started by addressing the level of HHV6-specific T cells in peripheral blood, against four antigens, U11, U39, U54 and U90. These antigens were chosen to represent antigens expressed immediately upon infection (U90) or present as part of the virion (tegument proteins U11 and U54, and viral glycoprotein U39), and being functional homologues of immunogenic HCMV antigens (IE1, pp150, pp65 and gB, respectively) 15,27. After overnight stimulation with whole antigen PepMixes^TM^, ICS was used to identify CD8+IFNγ+ and CD4+IFNγ+ T cells. In our panel of 30 healthy donors responses were very low or undetectable. CD8+IFNγ+ responses over 0.1% were only observed against U90 in six donors, and U54 in one donor, with median values of 0.01% for U90 and 0.00% for U11, U39 and U54. Levels of CD4+IFNγ+ cells were also low but overall had higher median values for 3 out of the 4 antigens (U11 0.00%, U90 0.01%, U39 0.01% and U54 0.02%). In contrast, significant levels of HCMV IFNγ+ CD4+ and CD8+ responses to comparable antigens were observed in HCMV seropositive donors within this group. Thus, confirming we are able to detect herpesvirus-specific responses in our donors directly *ex vivo*, and that the very low levels of HHV6B-specific response are a feature of the immune response to HHV6B in these donors. Indeed, the levels of HHV6 T-cell reactivity detected in peripheral blood in study is in agreement with previous publications using viral infected cell lysates 23,28–30 and more recently using a similar PepMix^TM^ stimulation approach 25,26. It is known that T cell immunity to HCMV increases with age 31. This has not yet been shown for HHV6B, and the age range of our cohort of donors, 22–63 years with a median of 39 years, was not sufficient to address this point. However, our study does indicate that the responses detectable in peripheral blood in the young (20–40 years) and middle aged (40–60 years) are very weak. Further detailed investigations will be required to determine the strength of HHV6B T cell responses in the elderly (>60 years). We subsequently showed using ELISPOT analysis that IFNγ+ T cell responses could be expanded by stimulation with PepMixes^TM^ *in vitro* followed by a short-term culture. Using this approach we did not differentiate between CD4+ and CD8+ responses; however, all 30 of the donors responded to at least one antigen, with most (57%) responding to three or all four. Based on the percentage of donors responding and median frequency of IFNγ+ T cells, we observed a hierarchy of immunodominance amongst these antigens, with U90 and U54 being co-dominant followed by U11 and then U39. This is in general agreement with a previous report where responses against five antigens (U11, U15, U54, U71 and U90) were analysed in 14 donors, and U90 was found to be the most immunogenic, followed by U14 and then U54 25. It was not clear if the hierarchy determined in our study was influenced by predominantly antigen-specific CD4+ or CD8+ T cells, or an even distribution of both. Although, when the PepMix^TM^ expanded cultures were analysed for the percentage of CD3+ CD4+ and CD3+ CD8+ cells, we found that for all four antigens the majority for cells were CD4+. Our analysis was limited to measuring total CD4+ and CD8+ T cells in the expanded population, rather than also determining antigen specificity, and it could be argued that the CD4/8 T cell ratio observed in the expanded population reflects a non-specific expansion maintaining the same approximate ratio that is present in PBMC. However, the mainly CD4+ T cell response was similar to that seen when PepMix^TM^ expanded cells were analysed for frequency CD3+CD4+ and CD3+CD8+ cells based on production of IFNγ after antigenic stimulation 25 and also in agreement with earlier reports 28–30,32. Regardless of the exact composition of the expanded antigen-specific T cells, our data strongly supports the inclusion of these HHV6B antigens, particularly U90 and U54, in T cell immunotherapeutic regimes.

Why the cellular immune response to this herpesvirus is so low in peripheral blood of latently infected individuals is unclear. It is likely that, in common with other herpesvirus 33,34, HHV6 will encode immunosuppressive proteins. To date, one HHV6 protein, U21, has been shown to interfere with the MHC Class I antigen processing and presentation pathway 35. HHV6 has also been shown to have immunomodulatory effects, including infection of CD4+ T cells and induction of Treg cells 36–38. These factors may influence the development of HHV6-specific T cell responses. The level of HHV6 reactivation may also play a role. We assume the healthy individuals in this study have a stable latent infection, and it may be that the levels of virus reactivation from latency are insufficient to stimulate generation of high numbers of T cells, while still controlling reactivation. It would be of interest to carry out serial analysis of selected donors over a period of time to measure levels of HHV6-specific T cells, in parallel to monitoring viral load. Analysis of T cell responses during primary infection would be of central interest. Higher levels of HHV6-specific T cells, than seen in healthy individuals, have been detected in a HSCT patient with active HHV6B reactivation 25, suggesting that active stimulation by replicating HHV6B can induce expansion of virus-specific T cells. A recent study by de Pagter et al. correlating HHV6 reactivation and levels of virus-specific T cells in patients after HSCT supports this. These authors report that in HSCT patients where HHV6 reactivation had been evident there was also an expansion of HHV6-specific T cells, both CD4+ and CD8+ 39. Thus, providing encouraging support for the application of HHV6-specific T cell therapy either by augmenting such responses by prophylactic treatment or by adoptive T cell therapy. Indeed, Leen and colleagues have recently reported the first successful use of HHV6-specific T cells in adoptive T cell therapy in a patient with high levels of HHV6 after HSCT 40. Thus, supporting the need for continued studies elucidating details of the immune response to HHV6.

Identifying the immunogenic viral antigens is an important first step for development of T cell immunotherapy. However, it is also important to map and characterise T cell epitopes within these viral antigens, particularly MHC Class I restricted peptides. Thus, we extended our study to begin identifying HHV6B CD8+ T cell epitopes. We focussed on donors known to respond to U90 and U11, and used an unbiased approach with regard HLA background. After stimulation with the relevant PepMix^TM^, we depleted CD4+ T cells and screened for peptide reactive CD8+ T cells using overlapping 15-mer libraries. We identified three putative novel CD8+ T cell epitopes in U90, termed PSK, NHC and LNI. These epitopes were predicted to be restricted through HLA-A29, B39 and A1 respectively. The B39-restricted NHC epitope sequence conforms to the motif of peptides known to bind to HLA-B*3901, which have a highly conserved His at position 2, an aliphatic anchor at position 9 41. The LNI peptide, predicted to be restricted through HLA-A1, gave a weak response in comparison to the original 15-mer, and as HLA-A1 tends to have acidic residues at position 3, rather than position 4 for this peptide 41, it is possible that the real epitope begins NID and may be a 10-mer. Thus, we have termed this LNI peptide as a hypothetical epitope. We also found responses in three donors against the previously identified HLA B40(60)-restricted epitopes FES and VEE in U90 25. We also identified the epitope LKT in U11, which was presented by target cells that were HLA-B37, C6 positive. It is known that there is a strong genetic linkage between B*3701 and C*0602 42. Thus, based on characterised binding motifs, with B37 requiring an acidic anchor residue at position 2 and C6 a basic anchor residue at position 2 41, and known antigenic peptides 43,44 we predict that LKT is restricted through HLA-C7. These HLA alleles predicted to present the novel putative HHV6B T cell peptides are present in the caucasian population at average frequencies for A1 14.07%, A29 3.01%, B39 2.03% and C6 9.62% 45, highlighting their therapeutic relevance. Importantly, T cells specific for these novel HHV6 immunogenic peptides were also shown to recognise HHV6B-infected cells. It should be noted that the T cell populations generated will no doubt contain other non-HHV6B specific T cells that could contribute to the recognition observed. However, we do not believe that this has any significant impact on the data presented. Low background levels of recognition were observed, as expected utilising autologous PHA blasts as target cells in such assays, and a high percentage of virus infection was observed in all cases. Overall this data supports the suitability of immunotherapeutic targeting of both U90 and U11 across a range of HLA backgrounds.

For the development and enhancement of adoptive T cell therapy approaches for diseases driven by HHV6B, a comprehensive understanding of the overall immune response to this virus and details such as target antigens and peptides, and HLA-restricting alleles is vital. The data presented in this report adds to and expands the existing information, and supports the on-going efforts to develop T cell immunotherapy for HHV6 reactivation in transplant settings.

## Materials and Methods

### Donors, cell lines and virus stocks

PBMCs were obtained from healthy adult volunteers, ranging in age from 22 to 63 years (median 39 years). The study was approved by the Liverpool Adult Research Ethics Committee and informed consent was obtained from all participating donors according to the Helsinki Declaration. PBMCs were isolated by Ficoll Hypaque density gradient centrifugation using Ficoll-Paque^TM^ Plus (GE Healthcare Biosciences AB, Little Chalfont, UK). Donors were HLA typed using a PCR-based protocol by the Clinical Immunology Tissue Typing Laboratory, Royal Liverpool and Broad Green Hospital Trust, Liverpool, UK. In most cases, HLA types were obtained for A, B and C alleles, and provided to two-digit resolution. PHA blasts were generated from PBMC by treatment with 5 μg/mL PHA and culturing in T cell media (RPMI 1,640 medium with 5% human AB serum, 2 mM glutamine, 50 IU/mL penicillin and 50 µg/mL streptomycin). HHV6B virus (strain Z29) was generated as culture supernatant from SupT1/Z-29 cells 46, obtained from the HHV-6 Foundation (Santa Barbara, CA, USA). Briefly, HHV6B-Z29 infected SupT1 cells were maintained for 6 days in RPMI 1,640 media supplemented with 10% FCS and 2 mM glutamine. Virus-containing supernatant was then harvested, centrifuged at 400*g* for 5 min to remove cells, before being filtered through a 0.45 μm Minisart® NML Syringe Filter (Sartorius UK Ltd., Epsom, UK). Virus containing supernatant was stored in single use aliquots at −80°C.

### Peptides

Single tube PepMixes^TM^, containing 15-mer peptides (overlapping by 11aa) spanning the whole protein for HHV6B antigens U11, U39, U54 and U90, HCMV antigens IE1, IE2, pp65 and gB, and BK polyomavirus VP1 were obtained from JPT technologies (Berlin, Germany). Individual 15-mer peptides, overlapping by 11aa, for HHV6B antigens U11 and U90, were provided by Dr Ann Leen (Baylor College of Medicine, Houston, USA). The individual U11 and U90 15-mer peptides were used to generate mini-pools of 13–17 peptides, such that there were 30 mini pools for U11 and 33 for U90, with each 15-mer represented in 2 mini pools 47. Individual 9-mer peptides for HHV6B U11 and U90 were purchased from Alta Bioscience Ltd. (Birmingham, UK). All peptides were synthesised at >70% purity, and stocks were dissolved in DMSO at 10 mg/mL and stored at −20°C

### Intracellular cytokine staining

PBMCs (5 × 10^6^/mL) were incubated with PepMixes^TM^ (final concentration 2 μg/ml) in a 5 ml polystyrene tube at 37°C for 2 h in T cell media, before addition of Brefeldin-A (10 μg/mL, Sigma, Gillingham, UK), and incubation continued for a further 14 h. Controls were unstimulated PBMC and PBMC stimulated with ConA (5 mg/mL). PBMCs were then washed twice with PBS at 4°C, pelleted and incubated with a PC5-conjugated anti-CD8 mAb (BD Biosciences, Oxford, UK) for 20 min at 4°C before being fixed with 4% paraformaldehyde. Cells were then permeabilised by incubation for 5 minutes with permeabilization buffer (eBioscience, Hatfield, UK), followed by staining with a FITC-conjugated anti-IFN-γ mAb (BD Biosciences) for 30 min at 4°C in the dark. Cells were then washed twice with PBS, resuspended in 0.5 mL PBS, before 0.5 × 10^6^ were analysed for CD8+, IFN-γ+ve cells. Data was acquired on a BD FACSCalibur using Cell Quest Software, and analysed using FlowJo software (Treestar).

### ELISPOT

ELISPOT analysis was carried out using the Human IFN-γ ELISPOT kit from MABTECH (Stockholm, Sweden). T cell populations were added to wells at known cell numbers in duplicate or triplicate in the presence of either PepMixes^TM^, peptide mini pools or individual peptides, where the final concentration of each peptide was 2 μg/mL. Plates were incubated for 16 h, before addition of secondary antibody and subsequent detection reagents. The number of spot forming cells (SFC) was then determined manually. Controls in ELISPOT included unstimulated cells (DMSO treated) and cells stimulated with the BKV VP1 PepMix^TM^.

### ELISA

ELISA for human IFN-γ was carried out using an IFN-γ ELISA Ready-Set-Go® set (eBioscience), according to the manufacturer's guidance. Supernatants from T cell assays were measured directly or diluted as appropriate. Absorbance was read using a Multiskan Spectrum microplate reader (Thermo Scientific, Hemel Hempstead, UK) using SkanIt software 2.2. The concentration of IFN-γ (pg/mL) in each sample was calculated against the standard curve.

### Generation of HHV6B-specific T cells lines

Polyclonal HHV6-specific T cell populations were generated as follows. PBMCs (2 × 10^6^) were incubated with PepMixes^TM^ (where the final concentration of each peptide was 2 μg/mL) or individual peptides at a final concentration of 10 μg/mL in 100 μL RPMI for 60 min at 37°C. Cells were resuspended in T cell media and seeded in a 24-well plate. T cell cultures were supplemented on day 3 with 100 IU/mL IL-2 (Sigma), which was replenished every 3 days. On day 10 onwards T cells were harvested, counted and used in subsequent assays. CD8-enriched polyclonal T cell lines were generated from 10 days polyclonal T cell cultures by depletion of CD4+ T cells using an EasySep™ Human CD4 Positive Selection Kit (STEMCELL, Manchester, U.K.) according to the manufacturer's instructions. The efficiency of CD8+ T cell enrichment was analysed by flow cytometry, and monitoring CD4+ depletion which ranged between 95% and 99%.

### Mapping T cell epitopes

Mapping of minimal T cell epitopes was carried out by direct stimulation of expanded T cell populations, which had been enriched for CD8+ T cells. PBMCs were reactivated with individual 15-mer peptides, enriched for CD8+ T cells at day 10, before being incubated with overlapping 9-mer peptides representing the 15-mer peptide and analysis by ELISPOT.

### Identification of HLA restriction element

Autologous, partially HLA-matched and HLA mis-matched PHA blasts or PBMCs were used as targets in T cell assays. Target cells were loaded with individual peptides (2 µg/mL) for 2 h in volume of 100 μL RPMI at 37°C, then washed 3 times with RPMI. Peptide-pulsed cells were then resuspended in T cell media at 1 × 10^5^ cells/mL and used as targets in a T cell assay. Target cells (1 × 10^4^ cells in 100 μL) were added in triplicate to wells of a 96-well V-bottom plate and 100 μl of T cells from short-term *in vitro* reactivations were added at an Effector-to-Target (E/T) ratio of 10:1. The effectors and targets were co-cultured overnight at 37°C, before 100 μL of supernatant was harvested and monitored for IFN-γ by ELISA.

### T cell recognition of HHV6B Z29-infected cells

PHA blasts (2 × 10^6^) in 500 μL T cell media were infected with 500 μL of HHV6B virus-containing supernatant for 90 min at 37°C. A further 1 mL T cell media was added and the infection continued. Six days after infection, virus-infected PHA blasts and comparable mock-infected cells were counted, resuspended in T cell media at 1 × 10^5^ cells/mL and used as targets in a T cell assay. Target cells (1 × 10^4^ cells in 100 μL) were added in triplicate to wells of a 96-well V-bottom plate and 100 μl of T cells from short-term in vitro reactivations were added at E/T ratios between 20:1 and 5:1. The effectors and targets were co-cultured overnight at 37°C, before 100 μL of supernatant was harvested and monitored for IFN-γ by ELISA. Infection of PHA blasts by HHV6B was confirmed by monitoring visually for cytopathic effects and by immunofluorescence staining with a mouse–antihuman HHV6B mAb (monoclonal antibody MAB8535, clone C3108-003; Chemicon). The HHV6B mAb was used at a dilution of 1:50 in PBS, and incubated with cells for 90 min at 37°C in 5% CO_2_, before being stained with a secondary antibody (Goat anti-mouse IgG-FITC). Slides were counter stained DAPI then analysed with a Nikon fluorescence microscope.

## Supporting Information

**Figure S1**: A representative example of the flow cytometry analysis of the frequency of CD4+ and CD8+ T cells in a PepMix^TM^ expanded T cell population. **Figure S2**: A representative example of the flow cytometry analysis of the efficiency of CD4+ T cell depletion from PepMixTM expanded T cellpopulation. **Figure S3**: Environmental applications of organoclays for sorption of phenol and its derivatives **Figure S4**: Identification of a T cell response to HLA-A29 restricted T cell peptide PSKSKKIKL in donor HD33. **Figure S5**:Identification of T cell responses to 15-­mer peptide 226 in donor HD10 and peptide 104 in donor HD49. **Figure S6**: Identification of T cell responses to HLA-­B40 restricted T cell peptides VEESIKEL and FESLLFPEL.
