# Supplementary material for: Identification of novel CD8+ T cell epitopes in human herpesvirus 6B U11 and U90
Source: Immun Inflamm Dis. 2015 Apr 28;3(2):118–31. doi: 10.1002/iid3.55 (PMC4444154; doi:10.1002/iid3.55)
Supplement: Supplementary file 1 [file iid30003-0118-sd1.pdf]

Supporting Information Figure 1

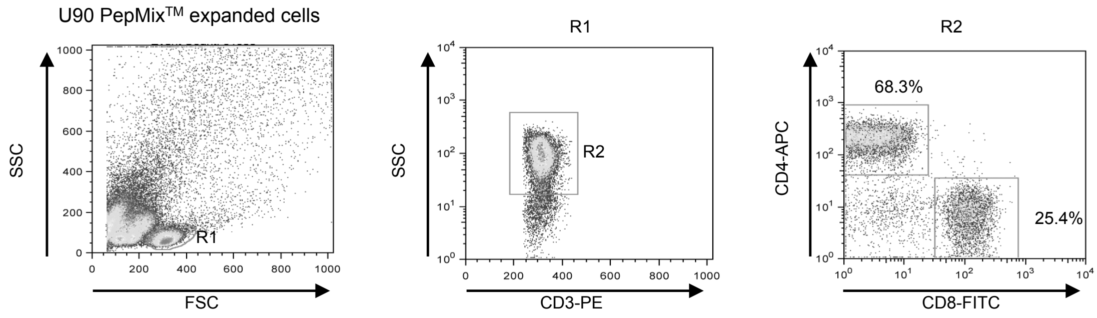

A representative example of the flow cytometry analysis of the frequency of CD4+ and CD8+ T cells in a PepMix™ expanded T cell population. PBMC from donor HD49 were stimulated with U90 PepMix™ for 10 days before cells were stained with anti-CD3-PE, anti-CD4-APC and anti-CD8-FITC, and analysed by flow cytometry. Lymphocytes were identified based on their forward- and side-scatter properties. The gated population (R1) was analysed for CD3+ cells, this population was then analysed for CD4+ and CD8+ T cells. Values indicated denote the percentage of CD4+ or CD8+ cells.

Supporting Information Figure 2

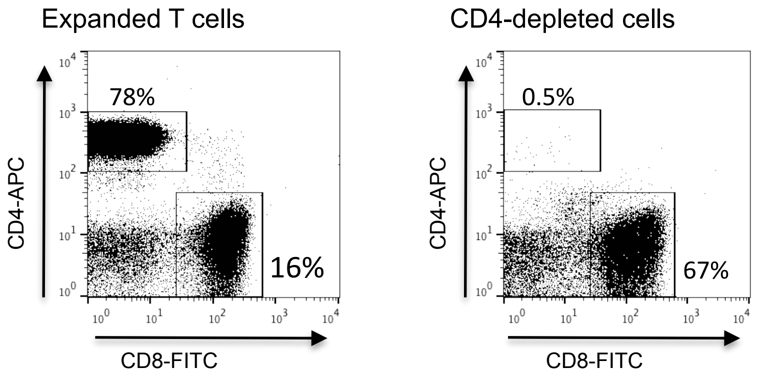

A representative example of the flow cytometry analysis of the efficiency of CD4+ T cell depletion from PepMix™ expanded T cell population. PBMC (from donor

HD53) were stimulated with U90 PepMix™ for 10 days before CD4+ T cells were depleted using EasySep™ Human CD4 Positive Selection Kit (STEMCELL, Manchester, U.K.). Expanded T cells before and after depletion were stained with anti-CD4-APC and anti-CD8-FITC, and analysed by flow cytometry. Lymphocytes were identified based on their forward- and side-scatter properties, and analysed for CD4+ and CD8+ T cells. Values indicated denote the percentage of CD4+ or CD8+ cells.

Supporting Information Figure 3

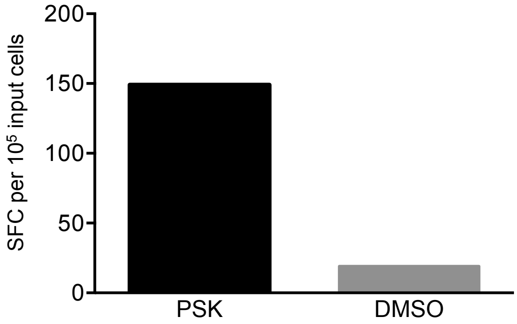

Identification of a T cell response to HLA-A29 restricted T cell peptide PSKSKKIKL in donor HD33. PBMC from donor HD33 (HLA A11, A29, B39, B44, C9, C16) were stimulated with the PSK peptide for 10 days. ELISpot analysis of the expanded T cell culture shows that donor HD33 contains T cells reactive to this HLA-A29 restricted 9-mer peptide from U90.

Supporting Information Figure 4

A

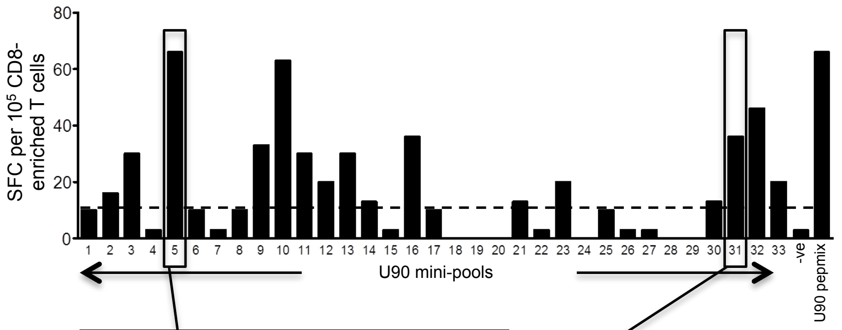

Pool	1	2	3	4	5	6	7	8	9	10	11	12	13	14	15	16	17
18	1	2	3	4	5	6	7	8	9	10	11	12	13	14	15	16	17
19	18	19	20	21	22	23	24	25	26	27	28	29	30	31	32	33	34
20	35	36	37	38	39	40	41	42	43	44	45	46	47	48	49	50	51
21	52	53	54	55	56	57	58	59	60	61	62	63	64	65	66	67	68
22	69	70	71	72	73	74	75	76	77	78	79	80	81	82	83	84	85
23	86	87	88	89	90	91	92	93	94	95	96	97	98	99	100	101	102
24	103	104	105	106	107	108	109	110	111	112	113	114	115	116	117	118	119
25	120	121	122	123	124	125	126	127	128	129	130	131	132	133	134	135	136
26	137	138	139	140	141	142	143	144	145	146	147	148	149	150	151	152	153
27	154	155	156	157	158	159	160	161	162	163	164	165	166	167	168	169	170
28	171	172	173	174	175	176	177	178	179	180	181	182	183	184	185	186	187
29	188	189	190	191	192	193	194	195	196	197	198	199	200	201	202	203	204
30	205	206	207	208	209	210	211	212	213	214	215	216	217	218	219	220	221
31	222	223	224	225	226	227	228	229	230	231	232	233	234	235	236	237	238
32	239	240	241	242	243	244	245	246	247	248	249	250	251	252	253	254	255
33	256	257	258	259	260	261	262	263	264	265	266	267					

Peptide 226: EANHCFINHFVPIKT

B

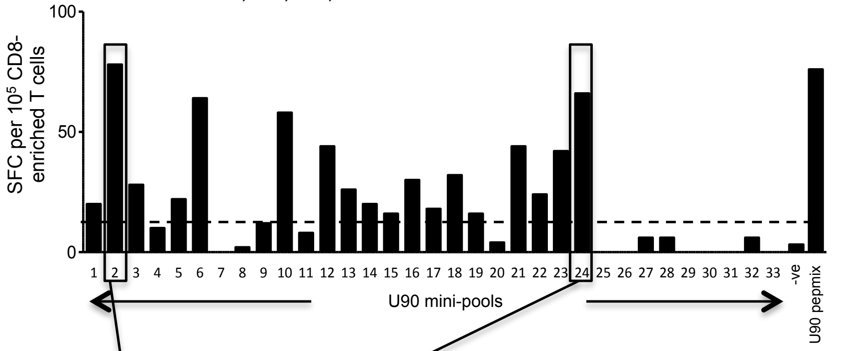

Pool	1	2	3	4	5	6	7	8	9	10	11	12	13	14	15	16	17
18	1	2	3	4	5	6	7	8	9	10	11	12	13	14	15	16	17
19	18	19	20	21	22	23	24	25	26	27	28	29	30	31	32	33	34
20	35	36	37	38	39	40	41	42	43	44	45	46	47	48	49	50	51
21	52	53	54	55	56	57	58	59	60	61	62	63	64	65	66	67	68
22	69	70	71	72	73	74	75	76	77	78	79	80	81	82	83	84	85
23	86	87	88	89	90	91	92	93	94	95	96	97	98	99	100	101	102
24	103	104	105	106	107	108	109	110	111	112	113	114	115	116	117	118	119
25	120	121	122	123	124	125	126	127	128	129	130	131	132	133	134	135	136
26	137	138	139	140	141	142	143	144	145	146	147	148	149	150	151	152	153
27	154	155	156	157	158	159	160	161	162	163	164	165	166	167	168	169	170
28	171	172	173	174	175	176	177	178	179	180	181	182	183	184	185	186	187
29	188	189	190	191	192	193	194	195	196	197	198	199	200	201	202	203	204
30	205	206	207	208	209	210	211	212	213	214	215	216	217	218	219	220	221
31	222	223	224	225	226	227	228	229	230	231	232	233	234	235	236	237	238
32	239	240	241	242	243	244	245	246	247	248	249	250	251	252	253	254	255
33	256	257	258	259	260	261	262	263	264	265	266	267					

Peptide 104: SICDLNIDPSESILL

Identification of T cell responses to 15-mer peptide 226 in donor HD10 and peptide 104 in donor HD49. U90 PepMix^{TM} expanded, CD4+ T cell-depleted, polyclonal T cell populations from (A) Donor HD10 and (B) Donor HD49 were screened by ELISPOT against the U90 15-mer mini-pools. For donor HD10, the intersect between mini-pools 5 and 31, peptide 226 (EANHCFINHFVPIKT), and for donor HD49, the intersect between pools 2 and 24, peptide 104 (SICDLNIDPSESILL) were selected for further analysis (see main text).

Supporting Information Figure 5

A

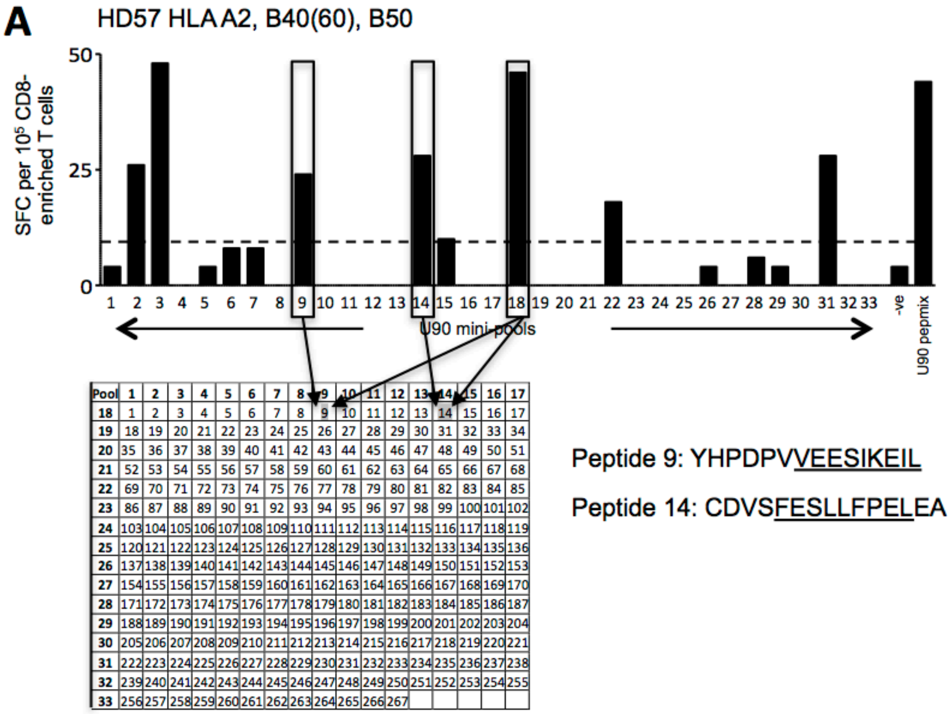

Pool	1	2	3	4	5	6	7	8	9	10	11	12	13	14	15	16	17
18	1	2	3	4	5	6	7	8	9	10	11	12	13	14	15	16	17
19	18	19	20	21	22	23	24	25	26	27	28	29	30	31	32	33	34
20	35	36	37	38	39	40	41	42	43	44	45	46	47	48	49	50	51
21	52	53	54	55	56	57	58	59	60	61	62	63	64	65	66	67	68
22	69	70	71	72	73	74	75	76	77	78	79	80	81	82	83	84	85
23	86	87	88	89	90	91	92	93	94	95	96	97	98	99	100	101	102
24	103	104	105	106	107	108	109	110	111	112	113	114	115	116	117	118	119
25	120	121	122	123	124	125	126	127	128	129	130	131	132	133	134	135	136
26	137	138	139	140	141	142	143	144	145	146	147	148	149	150	151	152	153
27	154	155	156	157	158	159	160	161	162	163	164	165	166	167	168	169	170
28	171	172	173	174	175	176	177	178	179	180	181	182	183	184	185	186	187
29	188	189	190	191	192	193	194	195	196	197	198	199	200	201	202	203	204
30	205	206	207	208	209	210	211	212	213	214	215	216	217	218	219	220	221
31	222	223	224	225	226	227	228	229	230	231	232	233	234	235	236	237	238
32	239	240	241	242	243	244	245	246	247	248	249	250	251	252	253	254	255
33	256	257	258	259	260	261	262	263	264	265	266	267					

Peptide 9: YHPDPVVEESIKEIL

Peptide 14: CDVSFESLLFPELEA

B

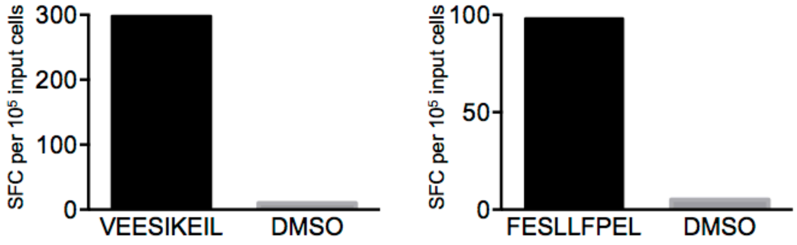

C

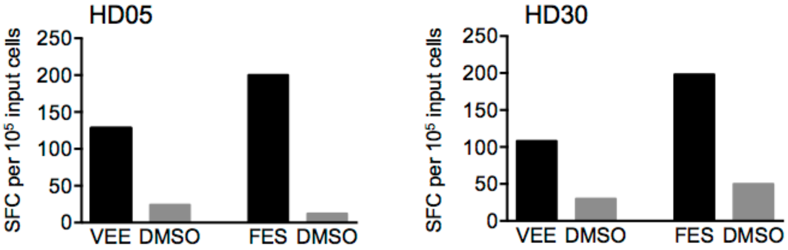

Identification of T cell responses to HLA-B40 restricted T cell peptides VEESIKEL and FESLLFPEL. (A) The graph shows the results of an ELISPOT screen, using the 15-mer mini-pools, of U90 PepMix^{TM} expanded polyclonal T cell populations from donor HD57 after depletion of CD4+ T cells. The 15-mer peptides representing the

intersect between mini-pools 9 and 18, peptide 9 (YHPDPVVEESIKEL), and pools 14 and 18, peptide 14 (CDVSFESLLFPELEA) were identified as positive responses in this donor. These peptides contain the previously identified HLA-B40 restricted peptides VEESIKEL and FESLLFPEL. (B) Subsequent ELISPOT screening of the expanded T cell populations showed that donor HD57 contained CD8+ T cells reactive to the VEE and FES peptides. (C) PBMC from two further HLA-B40 positive donors (HD05, HLA-A2, A24, B44, B40(60)) and HD30, HLA-A2, A32, B7, B40(60)) were stimulated *in-vitro* with the VEE and FES peptide, and the expanded T cells were screened by ELISPOT and shown to containing responses to both peptides.

Supporting Information Figure 6

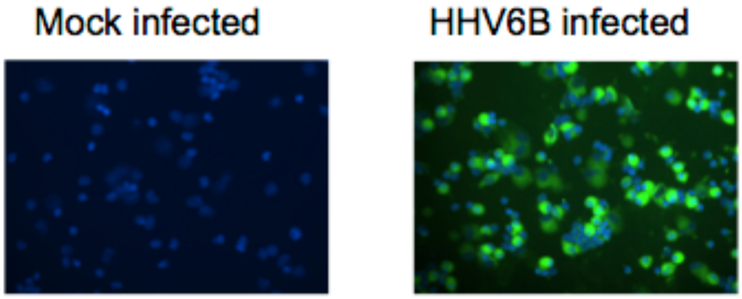

Immunostaining of HHV6 Z29 infected targets. Efficient viral infection of target cells in T cell assays was confirmed by immunofluorescent staining of HHV6B-infected and mock-infected cells with mouse-anti–human HHV6B mAb. The images shown are a representative example of the targets used in T cell assays.
